# A transient expression platform for rapid metabolic engineering of artemisinin biosynthesis in *Artemisia annua*

**DOI:** 10.3389/fpls.2026.1876067

**Published:** 2026-10-21

**Authors:** Fabio Pietrolucci, Vittoria Valesi, Elena Sgaravatti, Flavia Guzzo, Stefano Negri, Linda Avesani

**Affiliations:** 1 Department of Biotechnology, University of Verona, Verona, Italy; 2 PlantaRei Biotech Srl, Padova, Italy

**Keywords:** artemisinin, bioreactor, metabolic engineering, molecular farming, secondary metabolite, transient expression

## Abstract

Artemisinin, a sesquiterpene lactone produced in glandular trichomes of *Artemisia annua*, remains a key compound for artemisinin-based combination therapies against malaria. Although stable transformation has been widely used to enhance artemisinin production, rapid in planta systems for testing metabolic engineering strategies in *A. annua* remain limited. In this study, we developed a transient expression system based on vacuum agroinfiltration of whole *A. annua* plants, providing a fast and flexible tool to explore the engineering potential of the artemisinin pathway. Using GFP as a reporter, we established experimental conditions that support reproducible transient expression, identifying *in vitro*-grown plants as the most suitable material. We then assembled five key genes of the artemisinin biosynthetic pathway into a single multigene construct and tested its ability to redirect metabolism in both *Nicotiana benthamiana* and *A. annua*. While heterologous expression in *N. benthamiana* did not lead to detectable artemisinin-related metabolites, transient expression in *A. annua* produced a measurable, although temporary, shift in the specialized metabolome. At 2 dpi, targeted LC–HRMS analysis showed that artemisinin content increased from 0.086 ± 0.015 to 0.123 ± 0.012 mg/g fresh weight, while deoxyartemisinin and several putative sesquiterpenoid compounds also increased in relative abundance, suggesting that the introduced pathway genes influenced metabolic flux. These effects faded over time, revealing the strong endogenous regulatory constraints that shape artemisinin accumulation in its native plant host. Overall, this work establishes a rapid in planta platform for probing and modulating specialized metabolism in *A. annua*. Beyond artemisinin, it highlights the value of transient expression as a bridge between molecular design and plant-based production, supporting the broader vision of plants as dynamic biofactories for health-relevant natural products.

## Introduction

Malaria remains a global health challenge with approximately 282 million infections and 610,000 deaths still reported in 2024, primarily in African countries (World Health Organization, 2025). Due to the spread of resistance to conventional antimalarial drugs, the preferred treatment for malaria is currently represented by combination therapies based on artemisinin (ACTs), a sesquiterpene lactone featuring an endoperoxide bridge essential for its therapeutic efficacy (Ikram and Simonsen, 2017; O'Neill and Posner, 2004). Following its isolation in 1972 from the Chinese herbaceous plant *Artemisia annua*, artemisinin has become the most potent antimalarial compound, raising attention on *Artemisia* genus. For this discovery, which paved the way for “a novel therapy against malaria”, Youyou Tu was awarded the Nobel Prize in Physiology or Medicine in 2015 (Tu, 2011; Wang et al., 2019).

However, the yield of artemisinin from its natural source is very low, ranging from 0.02% to 0.8% of the dry weight (DW) of the leaves, occasionally reaching a maximum of 1.5% DW (Wani et al., 2021) depending on whether low-yielding or high-yielding *A. annua* cultivars are used (Yang et al., 2015). Due to the structural complexity of artemisinin, chemical synthesis is complicated and not cost effective (Schmid and Hofheinz, 1983; Zhu and Cook, 2012). Thus, alternative efforts have been undertaken over the past decades to meet the increasing clinical demand of artemisinin for ACTs, including biotechnological production in both native and heterologous hosts (e.g., yeast and tobacco) (Zhao et al., 2022; Zeinali et al., 2025). The mechanism of artemisinin production in *A. annua* plants has been well elucidated through the years, providing a solid basis to design metabolic engineering strategies (Brown, 2010; Ikram and Simonsen, 2017). The compound is produced in glandular trichomes (GTs), located on the surface of the *A. annua* aerial parts, mostly on the leaves, and accumulates in the subcuticular spaces of their apical cells (Olsson et al., 2009; Xiao et al., 2016). The first step in the biosynthetic pathway of artemisinin is catalyzed by the Farnesyl diphosphate synthase (FPS), which promotes, starting from two IPPs and one DMAPP units, the formation of the farnesyl diphosphate (FPP) (Brown, 2010; Wen and Yu, 2011). The 15-carbon FPP, which represents the common precursor of sesquiterpenoids, is then converted to amorpha-4,11-diene through the cyclization operated by the amorpha-4,11-diene synthase (ADS) (Mercke et al., 2000). Subsequently, the coordinated action of the cytochrome P450 monooxygenase (CYP71AV1) and its redox partner, the cytochrome P450 reductase (CPR), the amorpha-4,11-diene is oxidized first to artemisinic alcohol (AAOH), and then to artemisinic aldehyde (AAA) (Czechowski et al., 2016; Wang et al., 2011a). At this point, the biosynthetic pathway splits into two branches. Artemisinic aldehyde can be either oxidized directly to artemisinic acid (AA) by the aldehyde dehydrogenase 1 (ALDH1) activity or generate, under the action of artemisinic aldehyde Δ11(13) reductase (DBR2), dihydroartemisinic aldehyde (DHAAA), that is then converted to dihydroartemisinic acid (DHAA) by ALDH1 (Brown, 2010; Liu et al., 2016; Yang et al., 2015). Production of DHAA, which is considered the direct precursor of artemisinin, is the last enzymatic step in the metabolic pathway, since the final conversion in artemisinin happens through a photooxidation process in the subcuticular spaces of trichomes (Ikram and Simonsen, 2017; Zhao et al., 2022).

Metabolic engineering to boost artemisinin production in *A. annua* has evolved from relatively simple interventions, such as the overexpression of single key genes, to more sophisticated approaches, often applied in various combinations. These include the coordinated overexpression of multiple artemisinin biosynthetic genes, the knock-out of terpenoid competitive pathways, the modulation of transcription factors involved in pathway regulation, and the increase in glandular trichome density (Tang et al., 2022a; Li et al., 2024). The overexpression of genes codifying for key enzymes of the biosynthetic pathway, e.g. upstream genes of the terpenoid biosynthesis or final genes of the artemisinin pathway, has led to promising results, often reaching a 2/3-fold increase in artemisinin content in transgenic plants compared to controls (**Table 1**). Simultaneous overexpression of two or more key genes from the whole pathway seems to result in higher artemisinin accumulation and yield (Zhao et al., 2022). For instance, generation of transgenic plants overexpressing a combination of *ADS, ALDH1*, *CYP71AV1,CPR* led to the accumulation of 27 mg/g DW, a value 3.4-fold higher than the wild-type plants (Shi et al., 2017). More recently, the co-overexpression of *IDI*, *FPS*, *ADS*, *CYP71AV1, CPR*, *DBR2* with different subcellular localization allowed to produce a transgenic *A.annua* line accumulating 30 mg/g DW artemisinin, with a 232% increase compared to wild type plants (Qamar et al., 2024).

**Table 1:** Genetic engineering approaches applied to increase artemisinin (AN) content in *A. annua* plants.

Target gene(s)	AN content	AN increase*	Reference
*FPS*	10 mg/g DW	3-fold (+200%)	Chen et al., 2000
*FPS*	9 mg/g DW	+34%	Han et al., 2006
*HMGR*	n.p.	+23%	Aquil et al., 2009
*ADS*	1.2 mg/g DW	n.p.	Ma et al., 2009
*FPS*	6.71 mg/plant	3.6-fold (+260%)	Banyai et al., 2010
*HMGR*	1.73 mg/g DW	7.65-fold (+665%)	Alam and Abdin, 2011
*HMGR*	0.6 mg/g DW	+39%	Nafis et al., 2011
*HMGR*+*FPS*	9 mg/g DW	1.8-fold (+80%)	Wang et al., 2011b
*CYP71AV1*	0.98 mg/g FW	+38%	Shen et al., 2012
*DXR*; *CYP71AV1*+*CPR*	2.44 mg/g DW	2.7/3-fold (+170/200%)	Xiang et al., 2012
*FPS*+*CYP71AV1*+*CPR*	2.9 mg/g FW	3.6-fold (+260%)	Chen et al., 2013
*ADS*+*CYP71AV1*+*CPR*	15.1 mg/g DW	2.4-fold (+140%)	Lu et al., 2013
*HMGR*+*ADS*	12 mg/g DW	1.9-fold (+90%)	Alam et al., 2014a
*DBR2*	1.50-2.14 mg/g DW	1.59/2.26-fold (+59/126%)	Yuan et al., 2015
*FPS*+*ADS*	25 mg/g DW	2.5-fold (+150%)	Han et al., 2016
*ADS*+*CYP71AV1*+*CPR*+*ALDH1*	27 mg/g DW	3.4-fold (+240%)	Shi et al., 2017
*HMGR*+*FPS*+*DBR2*	32 mg/g DW	n.p.	Shen et al., 2018
*IDI*+*FPS*+*ADS*+*CYP71AV1*+*CPR*+*DBR2*	30 mg/g DW	+232%	Qamar et al., 2024

*AN increase in transgenic lines is reported relative to control plants, as cited by the authors (when fold change rather than percentage increase is reported, it has been converted to percentage as shown in brackets); n.p., values not provided by the authors.

In this work, transient expression was explored as a fast and flexible alternative to stable transformation (Krenek et al., 2015) to investigate the effects of genetic manipulation on the artemisinin biosynthetic pathway. Using GFP as a reporter gene, we first developed a protocol for transient expression in A. annua plants. We then used this protocol to study the effects of expressing five structural genes of the artemisinin biosynthetic pathway in both the model plant *Nicotiana benthamiana* and *A. annua*: farnesyl diphosphate synthase (*AaFPS*), amorpha-4,11-diene synthase (*AaADS*), cytochrome P450 monooxygenase (*AaCYP71AV1*), artemisinic aldehyde reductase (*AaDBR2*), and aldehyde dehydrogenase (*AaALDH1*).

## Materials and methods

### Plant material

*A. annua* plants cv. # 20931 F1, a high artemisinin yielding variety, were used in this work. Seeds have been provided from the East West Seed International Limited (Sai Noi, Sai Noi District, Thailand). Plants were grown from seeds both *in vitro* conditions in a growth chamber and in a greenhouse. *In vitro* plants were kept on Murashige and Skoog medium supplemented with 2% sucrose and exposed to a light/dark cycle of 18/6 hours at 24 °C. Plants grown in the greenhouse had a light/dark cycle of 16/8 hours with temperature between 21-30 °C and relative humidity of approximately 32-50%.

*N. benthamiana* plants aged 4–6 weeks were used in this study. Plants were grown from seeds in soil and maintained in a growth chamber under a 9 h light/15 h dark cycle at 24 °C.

### Cloning of artemisinin biosynthetic pathway genes

The CDS of the selected genes (*AaFPS*, GQ420346.1; *AaADS*, KR445687.1; *AaCYP71AV1*, DQ268763.1; *AaDBR2*, EU704257.1; *AaALDH1*, FJ809784.1) were retrieved from the literature and synthetized *de novo* through the Gene Art service (Thermo Fisher, Waltham, USA). The CDS were then assembled into the expression vectors by using the Golden Braid technology (Sarrion-Perdigones et al., 2013), as described below. To eliminate the presence of recognition sites for *BsaI* and *BsmBI*, the original CDS of *AaADS*, *AaCYP71AV1* and *AaALDH1* have been mutated, yet maintaining the original frameshift. All CDS were cloned into *pUPD2* after restriction with *BsmBI*. Following *E. coli* transformation and plasmid purification, the insertion was verified through *BsaI* restriction, PCR and sequencing. The CDS of *ADS* and *ALDH1* have been cloned from *pUPD2* into *pDGB3alpha1* generating the *pDGB3_alpha1.35S::ADS* and *pDGB3_alpha1.35S::ALDH1* plasmid. On the other hand, the CDS of *DBR2*, *CYP71AV1* and *FPS* have been cloned from pUPD2 inside *pDGB3_alpha2* vectors generating *pDGB3_alpha2.35S::DBR2*, *pDGB3_alpha2.35S::CYP71AV1* and *pDGB3_alpha2.35S::FPS* vectors. Subsequently, *pDGB3_alpha1.35S::ADS* and *pDGB3_alpha1.35S::ALDH1* have been combined with *pDGB3_alpha2.35S::DBR2* and *pDGB3_alpha2.35S::CYP71AV1* using, respectively, *pDGB3_omega1* or *pDGB3_omega2* as destination vectors. The vectors *pDGB3_omega1.35S::ADS.35S::DBR2* and *pDGB3_omega2.35S::ALDH1.35S::CYP71AV1* were obtained and then combined to generate *pDGB3_alpha1*.*35S::ADS.35S::DBR2.35S::ALDH1.35S::CYP71AV1* vector. Finally, the 4 genes vector obtained were combined with *pDGB3_alpha2.35S::FPS* to create a unique five genes expressing vector, *pDGB3_omega1.35S::ADS.35S::DBR2.35S::ALDH1.35S::CYP71AV1.35S::FPS*. Type IIS restriction enzymes were purchased by New England BioLabs (Ipswich, USA). Multipartite and binary assemblies of *pUPD2* or *pDGB3* vectors were performed according to developer’s instructions (https://gbcloning.upv.es/). For each assembly, the ligation product was transformed into One Shot TOP10^®^*E.coli* cells by heat-shock, which were then plated on LB medium with the proper concentration of antibiotic (25 mg/L for chloramphenicol or 50 mg/L for kanamycin or spectinomycin) and 0.5 mM IPTG and 40 mg/L XGal (all purchased by Promega) to allow blue/white screening of positive clones. Plasmid purification was carried out through the E.Z.N.A.^®^ Plasmid Mini Kit I (Omega Bio-Tek, Norcross, USA). The purified plasmids were checked for proper assembly by restriction and PCR analysis by using specific pairs of primers (**Table 2**).

**Table 2:** Primers used for the assembly of GoldenBraid expression vectors.

Primer name	5’-3’ sequence
pUPD2_forward	GCTTTCGCTAAGGATGATTTCTGG
pDGB3_reverse	CAGGGTGGTGACACCTTGCC
pDGB3_forward	GGTGGCAGGATATATTGTGG
pDGB3_reverse	GTTTTCAGATGACGGCTGCACT
P35S_forward	GCCGACAGTGGTCCCAAAGATG
T35S_reverse	GGGAACTACTCACATTAT
*AaADS*_forward	CCAAGGCCGTTATTGATGGCTG
*AaDBR2*_forward	CGTTGGGTACACGGATTACCCT
*AaDBR2*_reverse	GAACTTGCCCATCTTGTAGGCAG
*AaALDH1*_foreward	ATTTAGCACTCGACCGGGATGC
*AaALDH1*_reverse	GAACTTCCATTAGCTCCTGAGCTC
*AaCYP71AV1*_forward	AGCCACGATGCAAAGAAAGACTG
*AaCYP71AV1*_reverse	GGAAGTGGTCAGTGAGAGTGCC
*AaFPS*_reverse	CCTCCAGGTATGTTGTAGTCAAGC

*Agrobacterium tumefaciens* EHA105 competent cells were transformed using electroporation method. These were then plated on solid LB medium containing the proper antibiotics for selection (50 mg/L rifampicin + 50 mg/L kanamycin) and colonies were checked by colony-PCR to assess the presence of all the putative inserted genetic elements. For GFP expression a GoldenBraid expression vector, *pDGB3α1.P35S::eGFP::TNos*, has been used.

### Transient expression by agroinfiltration

Transformed *A. tumefaciens* colonies were independently inoculated in 5 mL of selective LB broth, incubated at 28 °C in agitation (180 rpm) overnight. From this inoculum 500 µL was then transferred in 500 ml of selective media and incubated at 28 °C until the required OD_600_ value was reached. The bacteria were precipitated at 3 000*x*g for 20 min, and the pellet was re-suspended in an equal volume of MMA solution (MES 10 mM pH 5.5-5.6, MgSO_4–_10 mM, acetosyringone 100 mM, Tween20 0.01%). After the bacteria re-suspension, the solution was incubated at room temperature for 2–3 hours. Agroinfiltration in *A. annua* plants was performed with the aid of a vacuum pump. Plants were submerged in the infiltration solution and a pressure of 90 mbar was applied inside the infiltration chamber for 15 min followed by 45 s of release.

*N. benthamiana* plants agroinfiltration was performed with a 2 ml syringe without needle by injecting the infecting solution on the abaxial page. After the procedure, plants grown in soil were returned to their greenhouse or growth chamber. *A. annua* plants grown *in vitro* were placed on a sterilized inert substrate soaked with MS medium.

During agroinfiltration procedure a *pDGB3α2.P35S::P19::Tnos* vector was co-infiltrated together with the genes of interest.

For both species, control plants were subjected to the same vacuum-agroinfiltration procedure using non-transformed *A. tumefaciens* EHA105.

### Sampling

Sampling of agroinfiltrated plants expressing GFP took place 7 days post infection (dpi) for both *A. annua* and *N. benthamiana* plants. For plants grown in greenhouse, both *A. annua* and *N. benthamiana* leaves sampled from each individual represented a biological replicate. For *A. annua* plants grown *in vitro* pooling technique was applied during the sampling procedure, with each biological replicate being constituted by a pool of 5 distinct whole plants.

For experiments with the artemisinin biosynthetic pathway genes, *in vitro* plants were sampled at 2, 4, and 6 dpi, as described above. In a separate experiment, additional samples were collected at 12, 24, and 36 hpi for early transcriptional and targeted artemisinin analyses. The early qRT-PCR dataset comprised three biological replicates per treatment and time point, except for the 24 hpi control group, for which one sample was not amplifiable and two biological replicates were retained. The targeted artemisinin dataset comprised three biological replicates for most groups; the 12 hpi 35S group and the 24 hpi control group comprised two biological replicates. Leaves from *N. benthamiana* plants were sampled following the same procedure. All tissues were immediately frozen in liquid nitrogen and stored at −80 °C until analysis.

### Fluorescence stereomicroscopy

The LEICA MZ 16 F (Leica Microsystem, Wetzlar, Germany) stereomicroscope was used to visualize the presence of eGFP expressed in leaf tissues at 7 dpi. The observation of the emitted fluorescence was carried out by illuminating with UV light, generated by the LEICA CLS 150 X photonic cold light source, a portion of agroinfiltrated leaf and using the GFP3 filter, characterized by an excitation wavelength of 480/40 nm and a block filter at 530/20 BP. LEICA DFC420 C camera, connected to the stereomicroscope allowed to obtain a virtual image of the expression of eGFP in the leaf.

### RNA extraction and qRT-PCR analysis

Total RNA was extracted from leaves of agroinfiltrated *A. annua* and *N. benthamiana* plants using TRIzol^®^ Reagent (Invitrogen) according to the manufacturer’s instructions. RNA quantity and purity were assessed by NanoDrop™ One Microvolume UV-Vis Spectrophotometer (Thermo Fisher), and samples not meeting quality criteria (Abs260/Abs280: 1.8–2.2; Abs260/Abs230: 1.7–2.2) were purified by LiCl precipitation. RNA integrity was verified by 2% agarose gel electrophoresis. Genomic DNA was removed using TURBO DNA-free™ DNase (Ambion), and cDNA was synthesized from 2 μg total RNA using SuperScript III Reverse Transcriptase (Thermo Fisher) with oligo(dT) primers. cDNA quality was confirmed by PCR amplification of a 114-bp fragment of the *AaActin1* gene.

Gene expression analysis of *AaFPS, AaADS, AaCYP71AV1, AaDBR2*, and *AaALDH1* was performed with the primer pairs listed in **Table 3** by quantitative real-time PCR using GoTaq^®^ qPCR Master Mix (Promega) and a 1:10 dilution of cDNA. *AaActin1* was used as the reference gene. A melting curve analysis (55–95 °C, 0.3 °C increments) was included. Amplification efficiency and standard error (SE) were calculated as described by Zenoni et al. (2020). The primers amplify the corresponding endogenous gene sequences and it was not possible to distinguish transgene-derived transcripts from endogenous transcripts. For the early dataset, Welch’s t-test followed by Šidák correction for the three time-point comparisons within each gene was used to compare the 35S and control groups.

**Table 3:** Primers used for expression analysis of artemisinin pathway genes.

Primer name	5’-3’ sequence
*AaADS*_rt_forward	GTCTGTCGAATGGGCTGTCT
*AaADS*_rt_reverse	CTTTCTTGCTCGGCCTTGTG
*AaALDH1*_rt_forward	GGACTTGCCTCAGGTGTATTCA
*AaALDH1*_rt_reverse	CATCCCGGTCGAGTGCTAAATA
*AaCYP71AV1*_rt_forward	AGATACTGAGGCAAACTGGTGG
*AaCYP71AV1*_rt_reverse	TAGTTAACCGAGCTCTCTTGCC
*AaDBR2*_rt_forward	TAAGGAACAAGTCGAGGGTTGG
*AaDBR2*_rt_reverse	GGGTTGGTACACTTGATGGGAT
*AaFPS*_rt_forward	TCATCTGCCCTTGGTTGGTG
*AaFPS*_rt_reverse	TTGCCCTCTGCGTGTATGAG

### Total soluble proteins extraction and quantification

Total soluble proteins (TSP) were extracted from about 100 mg of leaf samples grounded in liquid nitrogen. Frozen powder was homogenized with 300 μL extraction buffer (50 mM sodium phosphate pH 8.0, 20 mM sodium metabisulfite, 0.5% Tween-20) supplemented with Protease Inhibitor Cocktail (Sigma-Aldrich, St. Louis, USA) and then centrifuged at 30000*xg* for 40 min at 4 °C. For total soluble protein (TSP) quantification of leaf extracts Bicinchoninic Acid (BCA) assay (EuroClone, EMP014500) was used. A calibration line was generated using known quantities of bovine serum albumin (BSA) in a concentration range between 0 and 2 μg/μL and 1:10 diluted leaf protein extracts were analyzed following manufacturer’s instructions.

### Western blot analysis

For western blot analysis, protein leaf extracts were diluted to the same concentration, added with sample buffer 4x (1.5 M Tris/HCl pH 6.8, 3% SDS, 15% glycerol, 4% β–mercaptoethanol) to a final 1x concentration and then boiled for 10 min. TSP were then separated by SDS-PAGE using 12% polyacrylamide gel and transferred to a nitrocellulose membrane by electroblotting. Western Blot analysis was performed as reported by Gecchele et al., 2015 with some modifications. GFP was detected using the anti-GFP antibody ab290 (Abcam, Cambridge, United Kingdom) diluted 1:7500, followed by incubation with a secondary horseradish peroxidase-conjugated rabbit anti-mouse IgG, diluted 1:5000 (A6154, Sigma-Aldrich). The signal was detected using ECL Select western blotting detection reagent (GE Healthcare, Little Chalfont, United Kingdom). The ChemiDocTM system (Bio-Rad Laboratories, Hercules, CA, United States) was used to capture chemiluminescent signals.

### Metabolite extraction

For each sample, 100 mg of frozen powder was extracted in 1.5 mL of LC-MS grade methanol (Honeywell, Charlotte, USA). The samples were mixed vigorously for 30 s, sonicated at 40 KHz in an ultrasonic bath Sonica^®^ Ultrasonic Cleaner (SOLTEC, Milan, Italy) at 4 °C for 15 min and centrifuged (16000 rcf, 15 min, 4 °C). Supernatant was collected and conserved at -80 °C for further analyses.

### Metabolomics analysis

Methanolic extracts were diluted 1:10 with ultrapure water (Honeywell), passed through Minisart RC4 filters (0.2 μm pores) (Sartorious, Göttingen, Germany), and 5 μL was injected into the UPLC device. The analysis was carried out with the methods previously described (Negri et al., 2024). Briefly, the chromatography consisted of a separation in reverse phase on a BEH C_18_ column (Waters) with a mobile phase (A: H_2_O+0.1% *v/v* formic acid; B: acetonitrile) flushing at 0.35 ml/min under a gradient increasing the apolar component throughout the analysis. The Xevo G3-QqTOF (Waters) mass spectrometer, was equipped with an ESI source operating in positive mode. MS data were acquired in continuum over an m/z range of 50-1500. Selected samples were further analyzed in Fast-DDA mode to obtain MS/MS spectra and support metabolite annotation. A mixture of authentic standards and a quality control (QC) sample, prepared by mixing equal aliquots of selected extracts, were periodically injected throughout the randomized sample batches to assess instrument stability over the course of the analyses.

Chromatographic data from *N. benthamiana* samples were manually inspected using MassLynx (Waters) to search for metabolites from the artemisinin pathway with the support of the following authentic standards: artemisinin (AN, Cayman Chemical, Michigan, USA); arteannuin b (AB), artemisinic acid (AA), deoxyartemisinin (dAN; Biosynth, Bratislava, Slovakia); dihydroartemisinic acid (DHAA), artemisinic aldehyde (AAA; LGC Toronto Research Chemicals, Toronto, Canada); dihydoartemisinic aldehyde (DHAAA; TLC Pharmaceutical Standards, Newmarket, Canada).

Raw chromatograms of *A. annua* samples were processed with Progenesis QI software (Waters) to extrapolate m/z features of metabolites detected in the extracts. Chromatographic profiles were aligned against QC, and a peak picking intensity threshold of 700 was applied to remove background ions. Further filtering was performed by comparison with blank samples. Raw peak area values from the resulting matrix were normalized as a percentage of the total ion signal in each sample. Normalized data were Pareto-scaled and subjected to principal component analysis (PCA) using SIMCA (Sartorius). To identify the metabolites varying between groups across timepoints, non-scaled data were log_2_-transformed and visualized through Volcano plots (thresholds: -Log(p-value)>1.13; |log_2_FC|>0.26).

Annotation of specialized metabolites in the data matrix was supported by matching retention time, accurate m/z, isotopic distribution and MS/MS fragmentation patterns with those from an in-house library of about 1,500 plant authentic standards. Moreover, a*.sdf* library of specialized metabolites isolated from *A. annua* was retrieved from the LOTUS Natural Products database, fragmented in silico and matched against the experimental MS/MS data using Progenesis.

### Quantification of artemisinin in *A. annua* extracts

*A. annua* extracts were diluted 1:10 in methanol and subsequently mixed 1:2 (v/v) with an aqueous artemisinin-d_3_ solution (0.4 ng/µL), yielding an overall 20-fold dilution and a final internal-standard concentration of 0.2 ng/µL. Three microlitres were injected, corresponding to 0.6 ng artemisinin-d_3_ on-column. Artemisinin calibration standards contained 0.15, 0.30, 0.60, 0.90, 1.20, 1.50, and 2.00 ng artemisinin on-column and a constant amount of 0.60 ng artemisinin-d_3_. Quantification was based on the ratio between the integrated extracted-ion chromatographic peak areas of artemisinin and artemisinin-d_3_. Linear regression of the peak-area ratio against the amount of artemisinin injected yielded y = 1.0867x − 0.0408 (R^2^ = 0.9958). Artemisinin concentrations in biological samples were calculated from the regression equation and corrected for injection volume, overall dilution, and extraction ratio. Results were expressed as mg artemisinin g^-^¹ FW. Statistical differences between control and 35S plants at each time point were assessed by two-way ANOVA followed by Šídák’s multiple-comparisons test (p < 0.05).

### Statistical analysis

All statistical calculations were performed using the GraphPad Prism version 8 software.

## Results

### Transient gene expression in *A. annua* plants

Three parameters were evaluated to optimize transient expression in *A. annua*: growth conditions, plant age, and *A. tumefaciens* culture density.

*GFP* expression was first assessed in plants grown either *in vitro* or in greenhouse conditions at 4, 5, and 6 weeks of age, following agroinfiltration with an Agrobacterium suspension at OD600 = 0.8. Western blot analysis of total soluble proteins revealed detectable GFP levels in all *in vitro*-grown plants regardless of age (**Figure 1A**). In contrast, greenhouse-grown plants showed more variable expression, with the highest signal observed in 6-week-old plants, although not all samples were positive, indicating lower infiltration efficiency.

**Figure 1:**
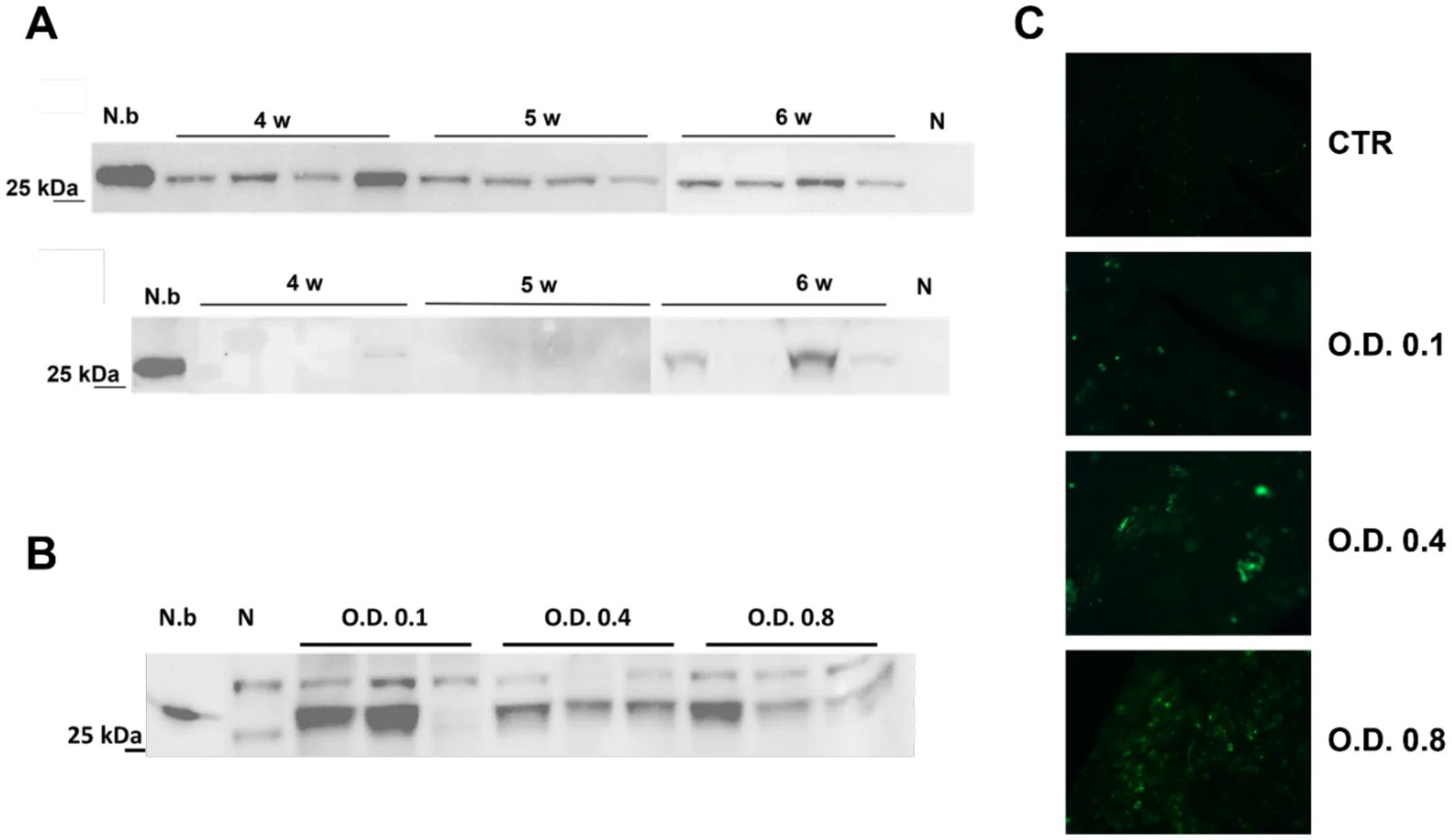
**(A)** GFP Western blot analysis of *A. annua* leaves from plants grown *in vitro* (upper line) or in greenhouse (lower line) and infiltrated with *A. tumefaciens* harboring *pDGB3α1.P35S::eGFP* at 4, 5 and 6 week age. 78.6 µg of TSP were loaded. N., negative control (*A. annua* plants infiltrated with EHA105 strain); N.b., *N. benthamiana* infiltrated with *A. tumefaciens* harboring *pDGB3α1.P35S::eGFP* used as positive control, 10 µg of TSP loaded. Side numbers indicate molecular mass markers in kDa. GFP is attended at 28 kDa. **(B)** GFP Western blot analysis of *A. annua* leaves from plants grown *in vitro* infiltrated with *A. tumefaciens* harboring *pDGB3α1.P35S::eGFP* at three different OD_600_ values. 112 µg of TSP were loaded for Aa plants; N., negative control, represented from *A. annua* plants infiltrated with EHA105 strain; N.b., *N. benthamiana* infiltrated with *A. tumefaciens* harboring pDGB3α1.P35S::eGFP used as positive control, 500 ng of TSP loaded. Side numbers indicate molecular mass markers in kDa. GFP is attended at 28 kDa. **(C)** GFP expression in leaves of *A.annua* plants infiltrated with *A. tumefaciens* harboring *pDGB3α1.P35S::eGFP* using 3 different OD_600_ value (0.1, 0.4 and 0.8) of bacterial solution. CTR, control group. Observation performed 7 dpi with LEICA MZ 16 F stereomicroscope. Pictures report abaxial surface of leaves. GFP expression is observed in cells of the epidermal layer of leaves.

Subsequently, different bacterial densities (OD600 = 0.1, 0.4, and 0.8) were tested in 6-week-old *in vitro*-grown plants. While the highest GFP signal was observed at OD600 = 0.1, more consistent results were obtained at OD600 = 0.4 and 0.8 (**Figure 1B**). Fluorescence stereomicroscopy further confirmed stronger GFP signal in plants infiltrated at OD600 = 0.8 (**Figure 1C**).

Based on these results, *in vitro* cultivation combined with an Agrobacterium density of OD600 = 0.8 was selected as the optimal condition for transient expression. No significant differences in GFP expression were observed among the tested plant ages; therefore, 6-week-old plants were used in subsequent experiments due to their higher biomass.

### Transient heterologous expression of multiple artemisinin biosynthetic genes in *N. benthamiana*

Five key genes of the pathway (*AaFPS*, *AaADS*, *AaCYP71AV1*, *AaDBR2*, and *AaALDH1*) were assembled into a single expression vector under the control of the CaMV 35S promoter (**Figure 2A**) used first in *N. benthamiana* and then in *A. annua*. RT-PCR analysis was performed to assess the successful transient expression of artemisinin biosynthetic genes in *N. benthamiana*,

**Figure 2:**
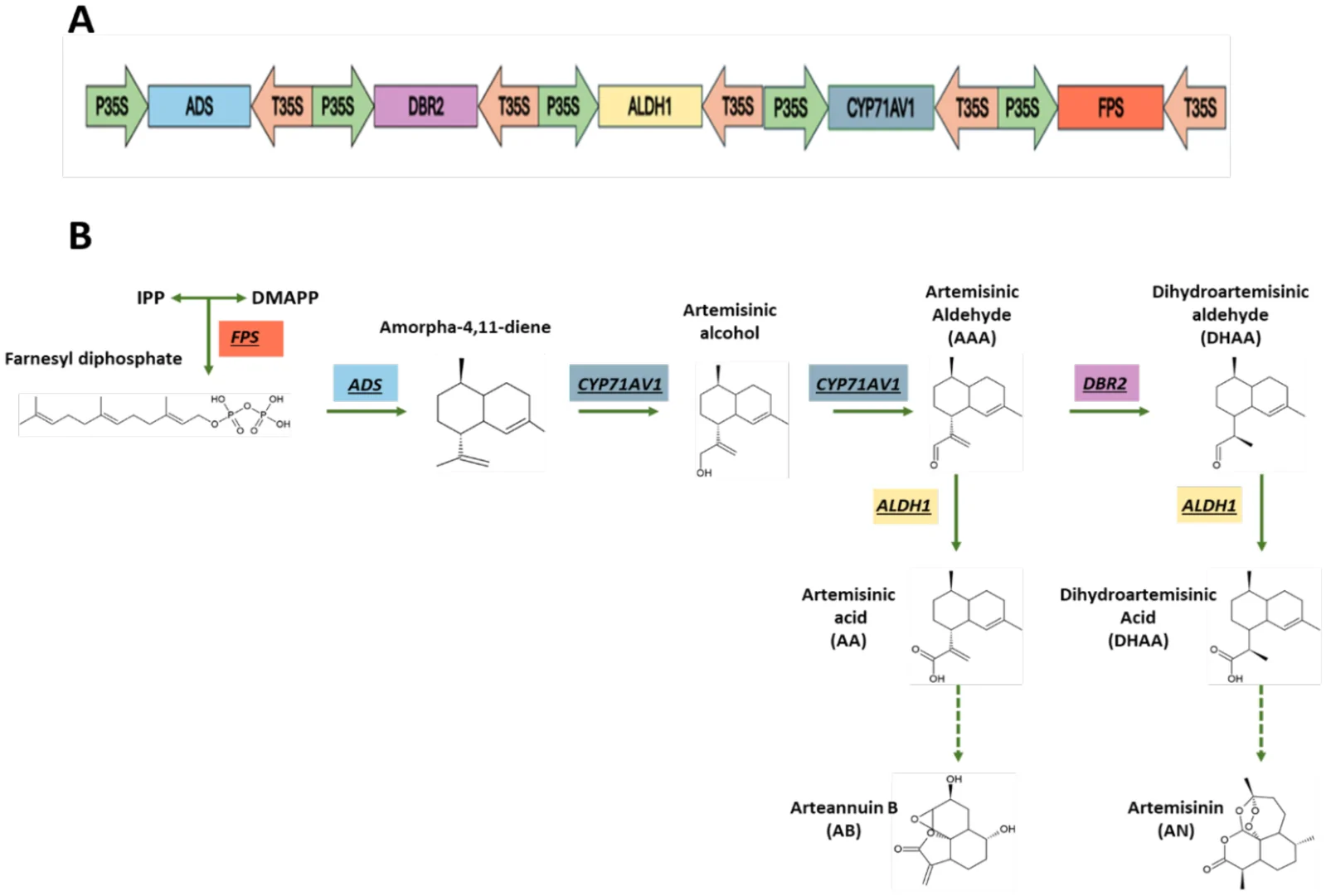
Five-gene construct **(A)** designed for the constitutive expression of structural genes from the artemisinin biosynthetic pathway **(B)**.

The analysis focused on *AaADS* and *AaFPS*, representing the first and last genes of the expression cassette, respectively. Expression of both genes was detected in all treatments at each time point, confirming the presence of the full construct (**Supplementary Figure S1**).

Methanolic extracts from transiently transformed *N. benthamiana* plants were pooled and analyzed by UPLC-ESI-HRMS. The m/z values of diagnostic ions [M+H^+^]^+^, [M+Na^+^]^+^, and [M+H^+^-H_2_O]^+,^ corresponding to the highest in-source generated adducts and fragments previously observed for authentic standards of artemisinin pathway metabolites (**Supplementary Figure S2**), were specifically searched in the chromatograms. However, neither artemisinin nor any of its precursors, up to the aldehydic intermediates, were detected in samples from agroinfiltrated plants.

### Transient expression of multiple artemisinin biosynthetic genes in *A. annua* plants

To evaluate the feasibility of modulating the artemisinin biosynthetic pathway (**Figure 2B**), the optimized transient expression protocol was applied to six-week-old *in vitro*-grown *A. annua* plants.

Plants were sampled over a 6-day time course at 2, 4, and 6 days post-infiltration (dpi), and gene expression levels were assessed by qRT-PCR (**Figure 3**). *AaFPS*, *AaDBR2*, and *AaALDH1* showed a similar expression pattern, with increased transcript levels at 6 dpi. Notably, *AaDBR2* exhibited a significant ~3-fold upregulation in treated plants compared to controls. Although not statistically significant, *AaFPS* and *AaALDH1* also displayed an increasing trend. In contrast, no consistent upregulation was observed for *AaADS* and *AaCYP71AV1*. Overall, high variability among biological replicates was detected, as indicated by the large standard deviations.

**Figure 3:**
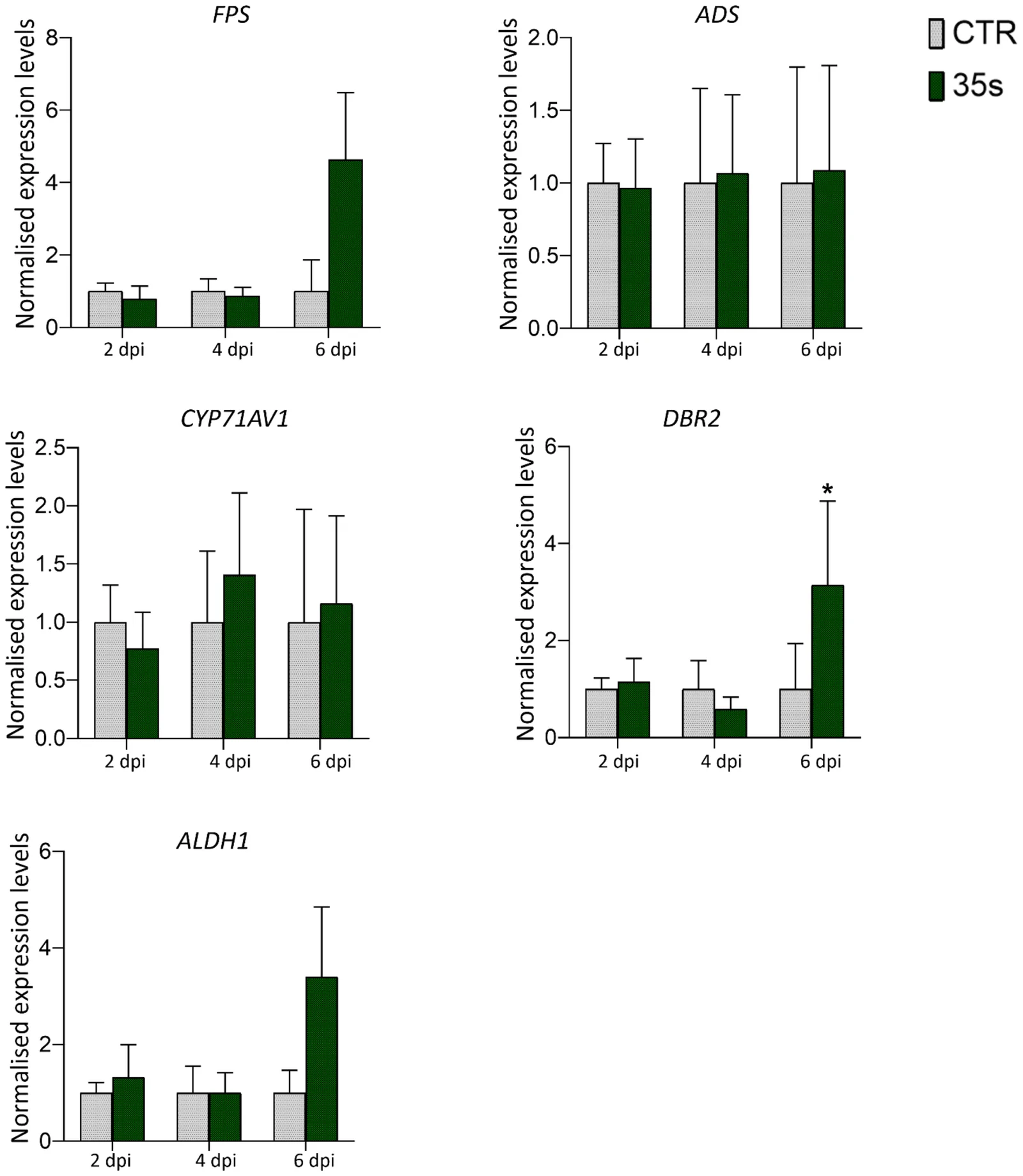
Expression analysis of artemisinin biosynthetic pathway genes in agroinfiltrated (35S) and control (CTR) *A. annua* plants at 2 dpi, 4 dpi and 6 dpi. Bars represent mean (n = 3) ± standard error. Student’s t-test was used to compare the differences in transcript level between transgenic lines and WT (*p<0.05).

### Variations in sesquiterpene levels in agroinfiltrated *A. annua* plants

The chromatographic profiles of *A.annua* extracts obtained through UPLC-ESI-HRMS in positive mode (**Figure 4A**) were processed to extrapolate a data matrix consisting of 792 m/z features. Peak area values were normalized to the total ion signal in each sample to investigate relative changes at the metabolome level of artemisinin pathway metabolites and of other non-volatile specialized metabolites eventually affected by the metabolic engineering approach used. Principal component analysis (PCA; **Figure 4B**) revealed that the main source of variability among samples was associated with the sampling time, which drove the separation along PC1 (24.6% of explained variance), showing a clear temporal progression of the metabolic profiles following the agroinfiltration procedure. Then, a separation between control and plants agroinfiltrated with the 35S construct was observed along PC2 (18.7% of explained variance), especially at 2 dpi (t1), whereas differences became less marked at later days (4 dpi, t2; 6 dpi, t3), suggesting a stronger impact at the earlier phase of the infiltration procedure. A certain degree of dispersion among biologic replicates was also observed, likely reflecting intrinsic biological variability of the *A. annua* plants, in both groups. Volcano plots were used to visualize the differences in metabolites across groups at three timepoints. We applied a log_2_fold change treshold of 0.26 to visualize statistically different m/z features (p-value<0.05 according to Student’s t-test) corresponding to an increase of at least 1.2-fold (*i.e.*, +20%) in the 35S group with compared to control. By looking at **Figures 4C–E**, the amount of m/z features characterizing each group progressively declined from t1 to t3, indicating that metabolome perturbation became very weak after 6 days post agroinfiltration. At t1 (**Figure 4C**), 67 m/z features characterized the 35S group, mostly eluting within the rt range 10–15 min of the analysis (**Supplementary Table S1**), with 18 of these features corresponding to the adducts and fragments of artemisinin. According to the most intense in-source generated ion [M+H^+^-H_2_O]^+^ detected with m/z 265.1452 showing a 0.42 log_2_fc, artemisinin (AN) increased of 34% in 35s compared to control. Moreover, also deoxyartemisinin (dAN) level was significantly impacted at t1 showing a 25% increase. As shown, in **Figure 4D**, AN and dAN were the only artemisinin pathway metabolites that varied significantly (p<0.05) in the 35s group, even though an increasing trend was in general observed at t1 in 35s plants also for arteannuin B (AB) and the acidic precursors (AA and DHAA). Despite detectable, the aldehydic intermediates (AAA and DHAAA), were filtered out during the generation of the data matrix because of their very low signal to noise ratio, making their evaluation not reliable for comparison among samples. Interestingly, among the specialized metabolites that increased in 35S plants at t1, one compound showed MS/MS fragment ions with identical m/z values to those of the sesquiterpenoid ilicic acid, but different fragment ion intensity ratios and a slightly shifted retention time (**Supplementary Figure S3**). This suggests that it may represent an ilicic acid closely related structural isomer. Furthermore, a series of m/z features corresponding to metabolites having a C_15_-based sesquiterpenoid skeleton were also detected at higher relative levels in the 35S plants. Despite the lack of authentic standards to confirm their identity, their fragmentation spectra showed a strong match with the *in silico*-predicted fragmentation patterns of metabolites previously isolated from *A. annua*, and displayed m/z values consistent with the [M+H^+^−H_2_O]^+^ adduct for the molecular formulas C_15_H_22_O_3_ and C_15_H_20_O_3_.

**Figure 4:**
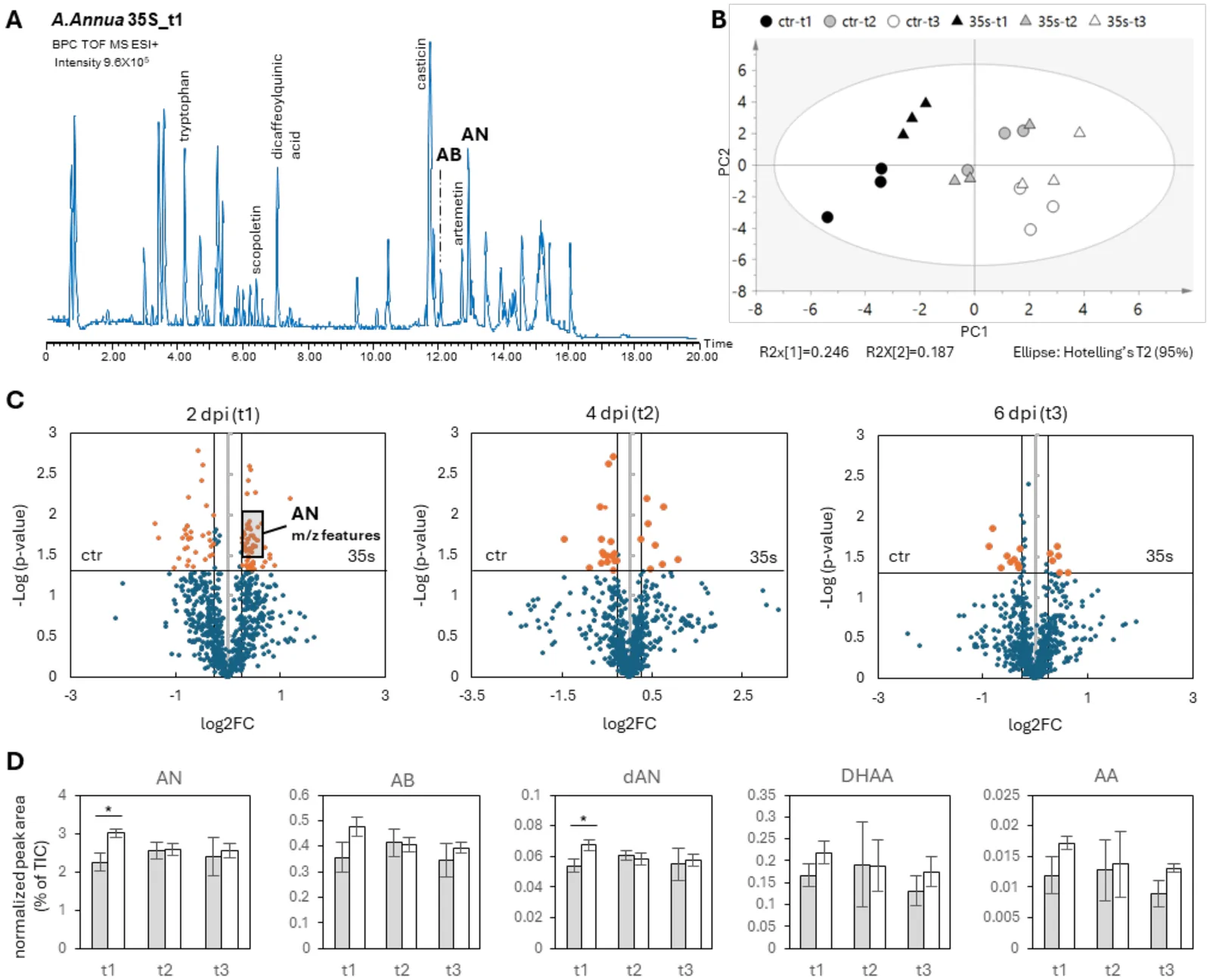
Untargeted metabolomics analysis of *A. annua* plants following transient overexpression of artemisinin biosynthetic genes. **(A)** Representative UPLC-ESI-HRMS chromatogram of a methanolic extract acquired in positive ion mode; **(B)** score plot from the unsupervised PCA of *A. annua* samples based on the m/z features extrapolated from the chromatograms. **(C)** Volcano plots comparing 35S and control samples at each time point; m/z features satisfying both statistical (p < 0.05) and fold-change thresholds (|log_2_FC| ≥ 0.26, corresponding to ≥ ± 20% change) are highlighted in orange; **(D)** Relative abundances of artemisinin (AN) and pathway-related metabolites, including arteannuin B (AB), deoxyartemisinin (dAN), dihydroartemisinic acid (DHAA), and artemisinic acid (AA), across time points. Data are presented as mean ± standard error (n = 3).

On the other hand, among the metabolites reduced in 35S compared to control, only the amino acids tryptophan and phenylalanine were annotated, while other m/z features remained unidentified, with most of them eluting within the polar range of the analysis.

### Early metabolomic and transcriptional dynamics following transient pathway overexpression

To further investigate the transient increase in artemisinin observed at 2 dpi and to clarify the temporal events preceding this metabolic response, an additional agroinfiltration experiment was performed, including earlier sampling points at 12, 24, and 36 h post-infiltration (hpi). These samples were used to assess both artemisinin accumulation and the early transcriptional response of the five overexpressed pathway genes.

Absolute quantification of artemisinin in all agroinfiltrated *A. annua* samples (**Figure 5**) was performed by LC–HRMS using deuterated artemisinin as an isotopically labelled internal standard. Artemisinin levels were comparable between CTR and 35S plants during the early time points. At 2 dpi, artemisinin content was significantly higher in 35S plants than in CTR plants (0.123 ± 0.012 vs. 0.086 ± 0.015 mg g^-^¹ FW; +44%; Šídák-adjusted p = 0.0385), providing direct targeted evidence of a transient increase in artemisinin abundance associated with the 35S treatment. Because this difference was detected specifically at 2 dpi, whereas the overall treatment effect and treatment × time interaction were not significant, it should be interpreted as a time-specific rather than sustained effect. Although the targeted assay confirms the absolute increase, the present data do not establish that it was caused exclusively by transgene-derived expression.

**Figure 5:**
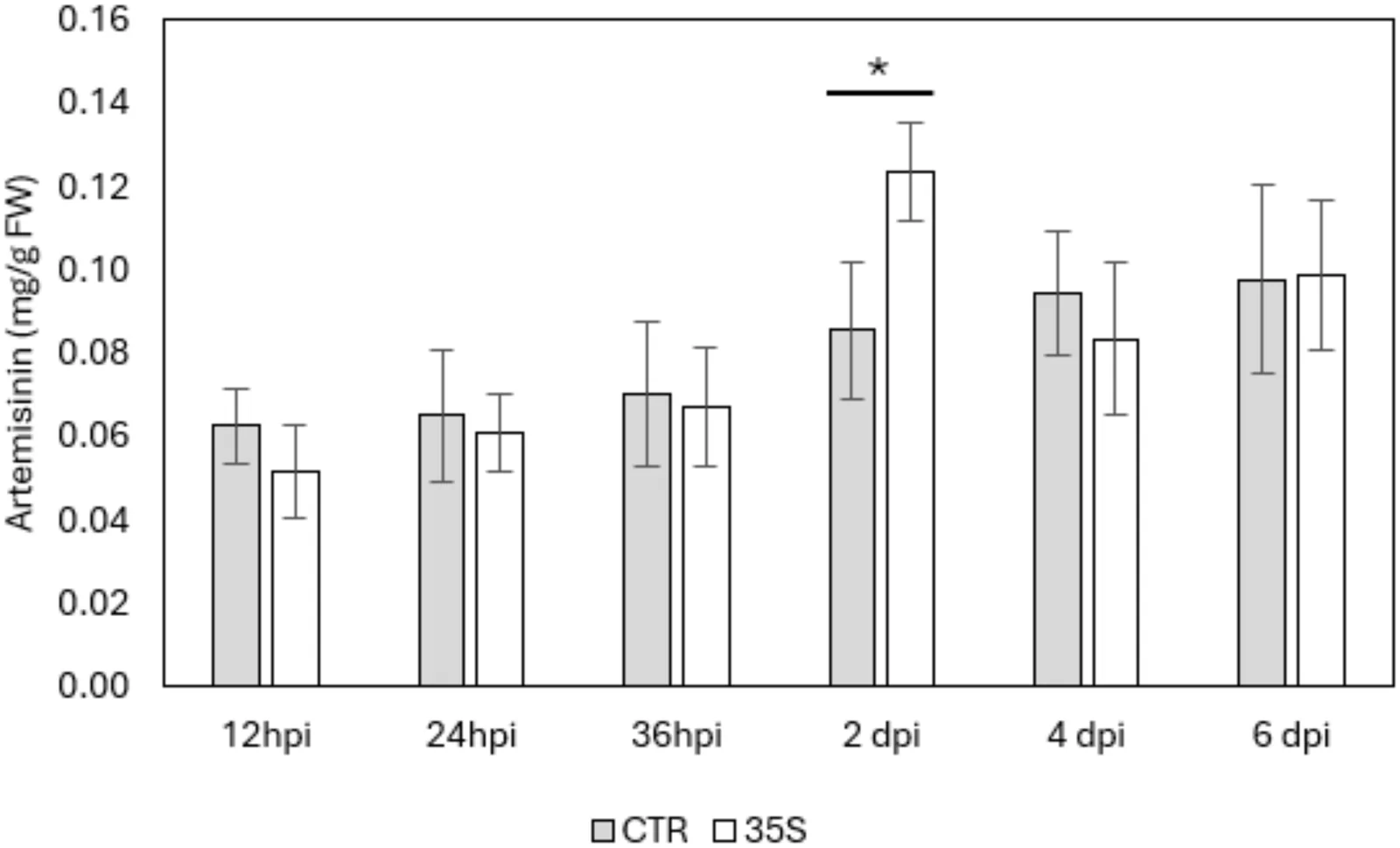
Absolute quantification of artemisinin in control and transiently treated *Artemisia annua* plants. Artemisinin content was quantified by LC–HRMS using deuterated artemisinin as an isotopically labelled internal standard in control (CTR) and 35S plants collected at 12, 24, and 36 h post-infiltration (hpi) and at 2, 4, and 6 days post-infiltration (dpi). Data are expressed as mg artemisinin g^-^¹ fresh weight (FW) and shown as mean ± SD. Sample sizes were n = 3 for most groups; the 12 hpi 35S group and the 24 hpi CTR group comprised n = 2. Statistical analysis was performed by two-way ANOVA followed by Šídák’s multiple-comparisons test between CTR and 35S plants within each time point. *p = 0.039.

Artemisinin levels were comparable between CTR and 35S plants during the early time points (12, 24 and 36 hpi), with mean concentrations ranging from approximately 0.062 to 0.074 mg g^-^¹ FW. At 2 dpi, artemisinin accumulation increased in 35S plants, reaching 0.123 ± 0.012 mg g^-^¹ FW compared with 0.086 ± 0.015 mg g^-^¹ FW in CTR plants, corresponding to an approximately 44% increase. Statistical significance was assessed by two-way ANOVA considering treatment (CTR vs 35S) and time as fixed factors, followed by Šídák’s multiple-comparisons test between CTR and 35S within each time point. The difference in artemisinin content at 2 dpi was significant after Šídák’s multiple-comparisons correction (p = 0.0385). At later time points, artemisinin levels were again comparable between the two groups, with no significant differences detected at either 4 or 6 dpi. Two-way ANOVA revealed a significant effect of time (p < 0.001), whereas neither the overall treatment effect nor the treatment × time interaction was significant.

To investigate whether an early transcriptional response preceded the increase in artemisinin observed at 2 dpi, the expression of *AaFPS*, *AaADS*, *AaCYP71AV1*, *AaDBR2*, and *AaALDH1* was evaluated at 12, 24, and 36 h post-infiltration (hpi) (**Figure 6**). Because the primers do not distinguish endogenous from construct-derived transcripts, these data demonstrate an early pathway-gene expression response associated with the agroinfiltration treatment but do not by themselves prove transgene-specific expression.

**Figure 6:**
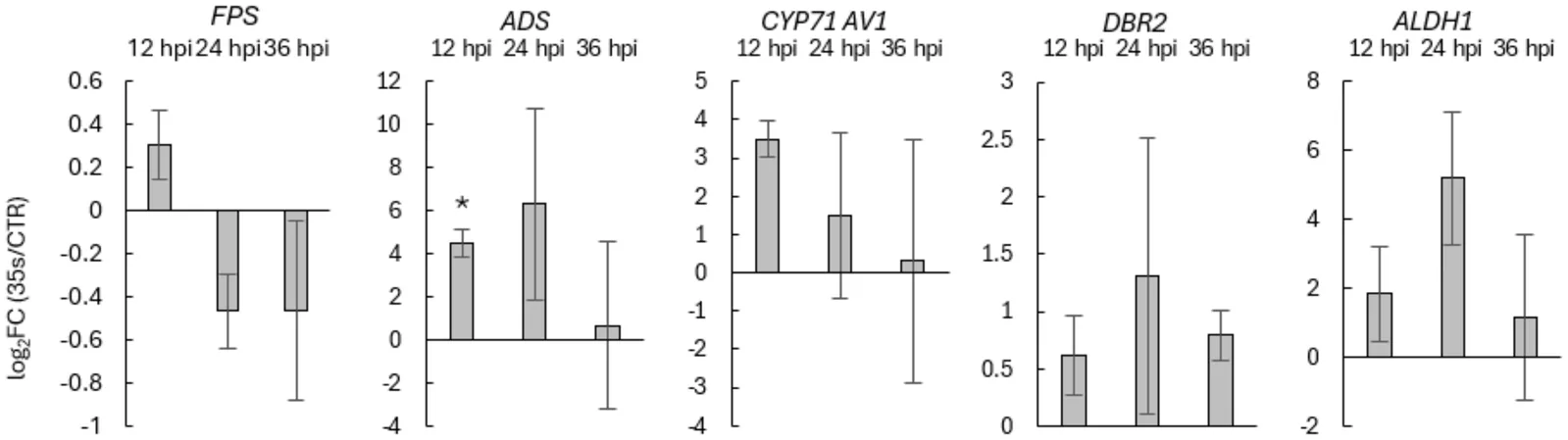
Early transcriptional response of artemisinin biosynthetic pathway genes following agroinfiltration with the multigene construct in *Artemisia annua*. Expression levels of *AaFPS, AaADS, AaCYP71AV1, AaDBR2*, and *AaALDH1* were evaluated at 12, 24, and 36 h post-infiltration (hpi). Data are expressed as log_2_ fold change (35S/CTR), calculated as 2^-^ΔΔCq using the mean ΔCq of the corresponding control group at each time point as calibrator. Bars represent mean ± SD. The number of biological replicates was n = 3 for most groups; the 24 hpi control group comprised n = 2 because one sample was not amplifiable. Statistical comparisons were performed using Welch’s t-test, followed by Šídák correction for the three time-point comparisons within each gene. *p<0.05.

The heatmap of row-wise z-scores calculated from −ΔCq values (**Supplementary Figure S4**) further highlighted the inter-replicate variability of the transient response. Several 35S samples displayed higher relative transcript accumulation than the corresponding controls, particularly for *AaADS*, *AaCYP71AV1*, *AaDBR2*, and *AaALDH1*, but the magnitude and persistence of the response varied substantially among biological replicates and time points. Overall, these results indicate an early but heterogeneous pathway-gene expression response associated with agroinfiltration with the multigene construct, with the clearest response occurring at 12 hpi for *AaADS*. This response preceded the significant increase in artemisinin content detected at 2 dpi, although the qRT-PCR data cannot establish that the measured transcripts were transgene-derived.

## Discussion

Despite extensive efforts to enhance artemisinin production in *A. annua*, transient expression approaches remain limited (Zhao et al., 2022). Most studies have relied on detached leaves (Elfahmi et al., 2014; Mirzaee et al., 2016; Nair et al., 2019), with only recent reports describing syringe-based agroinfiltration in seedlings (Li et al., 2021). Here, we establish a vacuum agroinfiltration protocol enabling transient gene expression in whole *A. annua* plants.

Our results identify *in vitro*-grown plants as the most suitable material, showing higher expression efficiency compared to greenhouse-grown plants. Similar observations have been reported in *V. vinifera* (Santos-Rosa et al., 2008). Leaf morphology is a key determinant of agroinfiltration efficiency (Wroblewski et al., 2005), and the complex architecture of *A. annua* leaves may limit infiltration (Li et al., 2021). The improved performance of *in vitro* plants may be explained by reduced stomatal functionality (Hazarika, 2006), facilitating bacterial entry, and potentially by structural differences such as increased intercellular spaces, as observed in other species (Bertini et al., 2018; Shekhawat and Manokari, 2018).

Bacterial density also played a critical role, with optimal and reproducible expression achieved at OD600 = 0.8, corresponding to the exponential growth phase. This is consistent with previous reports highlighting the importance of bacterial concentration in agroinfiltration efficiency (Hazarika, 2006; Alam et al., 2014b; Han et al., 2014; Li et al., 2021; Mirzaee et al., 2016).

Although the artemisinin biosynthetic pathway is well characterized, its regulation remains only partially understood. The transient system developed here provides a rapid platform to investigate this complexity.

### Metabolic engineering of the artemisinin pathway

The absence of detectable artemisinin-pathway metabolites in *N. benthamiana*, despite the detection of *AaADS* and *AaFPS* transcripts, suggests that transcription of the multigene construct alone was not sufficient to ensure functional pathway reconstruction in the heterologous host. Several non-mutually exclusive factors may account for this result, including inefficient translation of one or more transgenes, instability or improper folding of the corresponding proteins, and the lack of suitable endogenous cofactors or metabolic organization required for efficient pathway activity (Farré et al., 2014). In particular, CYP71AV1 activity may have been limited by the absence of a fully compatible cytochrome P450 reductase (Ting et al., 2013), while inefficient substrate channeling among pathway enzymes may have further constrained metabolic flux (Han et al., 2016). Thus, the negative result obtained in *N. benthamiana* highlights the importance of the host biochemical context for successful reconstruction of the artemisinin pathway.

In contrast, transient expression of the same multigene construct in its native host, *A. annua*, resulted in a measurable metabolic response. The metabolomics analysis was performed with the untargeted approach previously described (Negri et al., 2024), which has proven suitable for investigating artemisinin pathway metabolites alongside the medium-polar non-volatile components of the specialized metabolome.

To enable comparison across samples and capture relative changes in metabolite abundance, untargeted metabolomic data were normalized to the total ion signal. Accordingly, variations in artemisinin pathway metabolites were initially interpreted as changes in their relative abundance within the overall metabolite pool, rather than as absolute concentration changes. Importantly, the relative increase in artemisinin detected at 2 dpi was supported by targeted absolute quantification, which showed a 1.44-fold increase in the 35S group. This targeted result confirms a time-specific difference in artemisinin content but does not, in the absence of transgene-specific transcript measurements, prove that the difference was generated exclusively by the introduced genes. Hence, the observed difference should be considered exploratory.

It should also be noted that control plants received vacuum-agroinfiltration with non-transformed *A. tumefaciens* EHA105 alone, without co-infiltration of the P19 silencing suppressor used in the 35S treatment; the comparison therefore does not constitute a fully matched empty-vector/P19 control, and effects specifically attributable to P19 co-expression or to the vector backbone cannot be excluded.

At 12 hpi, *AaADS* and *AaCYP71AV1* showed the strongest relative expression response, with *AaADS* remaining significant after correction for multiple comparisons. However, because the primers used here do not distinguish transgene-derived from endogenous transcripts, the early response cannot be interpreted as direct evidence of transgene expression. At 24 hpi, the response became more heterogeneous among biological replicates, whereas by 36 hpi it was largely attenuated. These results provide a temporal association between an early pathway-gene expression response and the increase in artemisinin detected at 2 dpi. The causal contribution of the individual introduced genes remains to be established.

Additional constraints may arise from the use of multiple CaMV 35S promoters, which can trigger gene silencing (Thierry and Vaucheret, 1996; Bender, 2004; Eamens et al., 2008), as well as from promoter-specific variability in activity (Amack and Antunes, 2020). Gene silencing was not directly measured in the present study, so it remains a plausible explanation for the decline in the transient response but cannot be considered demonstrated. Notably, the 35S version used here has been reported to be less effective in *A. annua* compared to alternative constructs (Li et al., 2021).

Future strategies could focus on trichome-specific expression, where artemisinin biosynthesis naturally occurs (Tang et al., 2022b), or on targeting upstream regulatory networks. Indeed, transcription factors such as AabHLH1 and AaWRKY1 have been shown to enhance the expression of key pathway genes (Ji et al., 2014; Ma et al., 2009), suggesting that modulation of global regulatory circuits may be more effective than direct overexpression of structural genes.

## Conclusions and perspectives

We describe a vacuum agroinfiltration-based transient expression system for *A. annua* that enables rapid assessment of gene combinations and pathway perturbations. Agroinfiltration with a five-gene construct was associated with a moderate, time-specific increase in artemisinin and other putative sesquiterpenoids. However, the effects were transient, the response was heterogeneous, and the available qRT-PCR data do not distinguish transgene-derived from endogenous transcripts. In addition, because the control treatment did not include co-infiltration of the P19 silencing suppressor, the experimental design does not fully separate effects associated with P19 co-expression or the vector backbone. The system therefore provides a useful screening platform, while the causal contribution of individual introduced genes and the role of promoter silencing remain to be resolved.

Despite these limitations, this system may help prioritize gene combinations and regulatory strategies before stable transformation. Because glandular trichome density and structural maturity were not quantified in the *in vitro* plants, the extent to which this material functions as a representative artemisinin-producing cell factory remains an open question.

## Data Availability

The original contributions presented in the study are included in the article/**Supplementary Material**, further inquiries can be directed to the corresponding authors.

## Supplementary material

Supplementary Figure 1 RT-PCR of *AaADS* and *AaFPS* amplified from *N.benthamiana* cDNA obtained from control and 35S agroinfilitrated plants at 2, 4 and 6 dpi (respectively, t1,t2 and t3).

Supplementary Figure 2 LC-MS features of authentic standards of artemisinin-pathway metabolites. MS spectra recorded in the ESI^+^ full scan function are shown. AB, arteannuin B; AN, artemisinin; dAN, deoxyartemisinin; AA, artemisinic acid; DHAA, dihydroartemisinic acid; AAA, artemisinic aldehyde; DHAAA, dihydroartemisinic aldehyde.

Supplementary Figure 3 Comparison of MS/MS spectra of ilicic acid standard compound and of the putative structural isomer annotated in *A. annua* samples.

Supplementary Figure 4 Heatmap of early gene-expression responses following transient overexpression of artemisinin biosynthetic genes in *Artemisia annua*. Expression profiles of *AaFPS*, *AaADS*, *AaCYP71AV1*, *AaDBR2*, and *AaALDH1* are shown for individual biological replicates collected at 12, 24, and 36 h post-infiltration (hpi). Heatmap values correspond to row-wise z-scores calculated from −ΔCq values, so that positive z-scores indicate relatively higher transcript accumulation and negative z-scores indicate relatively lower transcript accumulation for each gene. Color code: -2, yellow; 0, white; +2, purple.

Supplementary Table 1 List of m/z features characterizing the plants transiently overexpressing the artemisinin pathway genes (35s) and the control plants at 2 dpi (t1). The threshold was set at the value of |log_2_FC|>0.26 (corresponding to about ±20%) and Log(p-value)>1.13 (corresponding to p-value <0.05).
