# Supplementary material for: A transient expression platform for rapid metabolic engineering of artemisinin biosynthesis in Artemisia annua
Source: Front Plant Sci. 2026 Oct 21;17:1876067. doi: 10.3389/fpls.2026.1876067 (PMC13732137; doi:10.3389/fpls.2026.1876067)
Supplement: Supplementary file 1 [file SupplementaryFile1.docx]

**Supplementary Figures**

**Figure S1.** RT-PCR of *AaADS* and *AaFPS* amplified from *N.benthamiana* cDNA obtained from control and 35S agroinfilitrated plants at 2, 4 and 6 dpi (respectively, t1,t2 and t3).

**Figure S2**. LC-MS features of authentic standards of artemisinin-pathway metabolites. MS spectra recorded in the ESI^+^ full scan function are shown. AB, arteannuin B; AN, artemisinin; dAN, deoxyartemisinin; AA, artemisinic acid; DHAA, dihydroartemisinic acid; AAA, artemisinic aldehyde; DHAAA, dihydroartemisinic aldehyde.

**Figure S3.** Comparison of MS/MS spectra of ilicic acid standard compound and of the putative structural isomer annotated in *A. annua* samples.

**Figure S4**. Heatmap of early gene-expression responses following transient overexpression of artemisinin biosynthetic genes in *Artemisia annua*. Expression profiles of *AaFPS*, *AaADS*, *AaCYP71AV1*, *AaDBR2*, and *AaALDH1* are shown for individual biological replicates collected at 12, 24, and 36 h post-infiltration (hpi). Heatmap values correspond to row-wise z-scores calculated from −ΔCq values, so that positive z-scores indicate relatively higher transcript accumulation and negative z-scores indicate relatively lower transcript accumulation for each gene. Color code: -2, yellow; 0, white; +2, purple.
